# Establishing a Diagnosis of Pulmonary Sarcoidosis

**DOI:** 10.3390/jcm12216898

**Published:** 2023-11-02

**Authors:** Jan C. Grutters

**Affiliations:** 1 ILD Center of Excellence, Department of Pulmonology, St. Antonius Hospital, 3435 CM Nieuwegein, The Netherlands; j.grutters@antoniusziekenhuis.nl; 2 Division of Heart and Lungs, University Medical Center Utrecht, 3584 CX Utrecht, The Netherlands

**Keywords:** pulmonary sarcoidosis, diagnosis, working diagnosis, multidisciplinary team discussion

## Abstract

Pulmonary sarcoidosis is the most prevalent manifestation of sarcoidosis and the commonest diagnosis in clinics for ILD. Due to the lack of a simple and reliable test, making the diagnosis is often challenging. There are three criteria that must always be considered: (1) compatible clinical presentation; (2) evidence of granuloma formation (usually non-caseating); and (3) exclusion of alternative causes of granulomatous disease. There are various tools available for diagnosis, amongst which serum biomarkers like sACE and sIL-2R, HRCT, BAL, EBUS/EUS and sometimes bronchoscopic or surgical lung biopsy are most contributive. However, the degree of invasiveness of the applied test and associated risk to the patient must be weighed against management consequences. In specific situations (e.g., presentation as Löfgren’s syndrome) or when there is high suspicion based on HRCT in the context of supportive clinical findings, it might be justifiable to decide on a “working diagnosis of sarcoidosis” and to refrain from further invasive procedures for the patient. This should, however, preferably be agreed upon after discussion in an experienced multidisciplinary team and requires close follow-up of the patient. In general, it is advisable to always maintain a healthy dose of skepticism when making the diagnosis of sarcoidosis, especially when the clinical course of disease gives rise to this.

## 1. Introduction

Pulmonary sarcoidosis is the most common manifestation of sarcoidosis and the most frequent established diagnosis in the group of interstitial lung diseases (ILD). Clinically, suspicion usually arises through finding of intrathoracic lymph node enlargement and/or diffuse nodular lung disease. Although HRCT is the cornerstone for diagnosis, its imaging findings are currently not considered diagnostically sufficient. Sarcoidosis has many lookalikes and currently remains a diagnosis by exclusion. Moreover, so far, no international agreement on the diagnostic approach has been brought about. Furthermore, usability and availability of diagnostic tools vary around the word. All in all, this makes establishing a diagnosis challenging.

## 2. Aim of Article

The aim of the article is to give an overview of the diagnostic criteria, differential diagnosis, clinical presentations, and approach to the diagnosis of sarcoidosis. Secondly, the value of the currently available tools for diagnosis is discussed. Further, the value of multidisciplinary team discussion and the concept of likelihood and working diagnosis are discussed. Finally, the reader is provided with a diagnostic algorithm based on the recent literature and experience of the author, along with future perspectives.

## 3. Definition of Disease

Sarcoidosis is defined as multisystem immune-mediated disease of unknown cause, pathologically characterized by non-caseating granuloma formation in various organs or tissues throughout the body. It commonly affects young and middle-aged adults and usually presents with bilateral hilar lymphadenopathy and/or pulmonary infiltration but can also manifest with ocular manifestations or skin lesions, and the liver, spleen, lymph nodes, salivary glands, heart, nervous system, muscles, bones, and other organs may also be involved [1,2].

## 4. Diagnostic Criteria

Three major criteria must be met to make a diagnosis of sarcoidosis: (1) a compatible clinical presentation; (2) findings of non-caseating granulomatous inflammation in one or more tissue samples; and (3) the exclusion of alternative causes of granulomatous disease or diseases capable of producing a similar clinical picture [2]. Recently, a multidisciplinary international panel of experts in sarcoidosis constructed clinically important questions related to diagnostic testing for sarcoidosis and performed systematic review of the evidence [3]. One strong recommendation, thirteen conditional recommendations, and one best-practice statement were formulated, of which the majority relate to screening for extra-pulmonary disease in patients with an established diagnosis of sarcoidosis. A summary of the recommendations related to lymph node sampling in patients suspected of pulmonary sarcoidosis is provided in Table 1. Unfortunately, all evidence was of very low quality.

## 5. Challenges in Diagnosis

A number of challenges in accomplishing a diagnosis of sarcoidosis are to be addressed.

First, there is significant heterogeneity in manifestations of the disease, which are often referred to as clinical phenotypes. There are not only various types of pulmonary involvement but also many extrapulmonary manifestations, and on top of that, many combinations of both are possible in sarcoidosis.

The GenPhenReSa (Genotype–Phenotype Relationship in Sarcoidosis) project, a European multicenter study, was designed to map in detail multi-organ involvement in over 2000 European sarcoidosis patients. The study found five distinct clusters according to predominant organ involvement: (1) abdominal organ involvement, (2) ocular–cardiac–cutaneous–central nervous system disease involvement, (3) musculoskeletal–cutaneous involvement, (4) pulmonary and intrathoracic lymph node involvement, and (5) extrapulmonary involvement [4]. Not surprisingly, lung involvement was 100% in cluster 4 (largest, represented 64% of patients) but also high (>90%) in clusters 1–3. Cluster 5 (6% of patients) showed only around 10% lung involvement. These data not only show the dominance of pulmonary involvement in sarcoidosis but also illustrate well the heterogeneity that challenges daily clinical practice.

Secondly, the defined multisystemic nature of the disease is clinically not always evident. It is well recognized from daily practice in specialized centers that isolated single-organ involvement may occur, especially in cardiac sarcoidosis. Also, other single-organ manifestations with strong suggestion of sarcoidosis may sometimes manifest without clinical evidence of a second organ’s involvement, leading to fundamental discussion about whether the diagnosis of sarcoidosis is acceptable [5,6].

Thirdly, the diagnosis of sarcoidosis needs exclusion of other causes of granulomatous disease such as tuberculosis, fungal infections, and organic and inorganic exposure-related ILDs such as hypersensitivity pneumonitis and berylliosis. Also, there are other rare diseases that might need consideration in proper context. Differential diagnosis of sarcoidosis is further discussed elsewhere. It should be noted that the likelihood of certain differential diagnosis of (pulmonary) sarcoidosis will differ around the world.

Finally, the diagnosis of sarcoidosis can never be 100% sure: It is a diagnosis of exclusion, and this cannot be accomplished with complete confidence. The diagnosis requires clinic-radiographic findings compatible with sarcoidosis, histologic or cytological confirmation of granulomatous inflammation, exclusion of known causes of granulomatous disease, and presence of disease in at least two organs or tissues. The end result of the diagnostic evaluation for sarcoidosis is neither a definitive diagnosis nor an exclusion of the diagnosis but rather a confident likelihood of the disease. In this light, a recent BTS clinical statement on pulmonary sarcoidosis raised the issue that decisions made by individual patients to decline bronchoscopy when there is a highly probable but not definite clinical diagnosis should be supported in most cases, with careful subsequent monitoring [7].

## 6. Clinical Presentation

Onset and type of symptoms of pulmonary sarcoidosis can vary largely. Most patients will manifest with gradual onset (symptoms present over months, sometimes years; can be progressive but not necessarily). Symptoms can be respiratory (e.g., ongoing cough or dyspnea on exertion) but also non-respiratory or combinations (e.g., fatigue, which can be the dominant-presenting symptom in pulmonary sarcoidosis). Because the presenting symptoms of sarcoidosis are not specific for the disease, the primary care physician is usually the first health care provider to assess the patient. If a patient with respiratory symptoms does not improve on (empirical) treatment for more common diseases like bronchitis, asthma, or chronic obstructive disease, it is usually through chest imaging, revealing signs of lymph adenopathy and/or diffuse lung disease, that referral to a medical specialist will takes place [8,9]. Also, a probably substantial but not specifiable proportion of sarcoidosis will remain asymptomatic and might only be found by chance, e.g., during medical examination or by self-referral body-screening services. The presenting symptoms of sarcoidosis will be discussed elsewhere in this issue.

Although most patients will manifest with gradual onset, a small subgroup of patients will present with symptoms of acute/subacute onset. These symptoms can be either directly related to sarcoidosis or indirectly, i.e., due to secondary complications. A well-known acute clinical manifestation of pulmonary sarcoidosis is Löfgren’s syndrome. Besides acute onset of disease, most commonly with fever, this syndrome is characterized by bilateral hilar lymphadenopathy, erythema nodosum, and/or ankle arthritis or marked periarticular inflammation of the ankles [10]. Another rare subtype of sarcoidosis, usually with acute presentation, is uveoparotid fever, also known as Heerfordt(–Waldenstrom)’s syndrome. This syndrome is charactered by a combination of facial palsy, parotid gland enlargement, and uveitis and is associated with low-grade fever [11]. In the majority of cases, hilar lymph node and/or lung involvement of sarcoidosis are also observed [12].

Occasionally, acute presentations can be caused by complications related to pulmonary sarcoidosis, such as pneumothorax, pneumonia, or pulmonary embolism [13]. These complications are, however, extremely rare and not regarded a characteristic course and onset of the disease.

## 7. Differential Diagnosis

After presentation of a patient with symptoms and/or signs on imaging that could be compatible with pulmonary sarcoidosis, various other diagnoses need to be considered, especially infectious diseases and malignancy like lymphomas. The differential diagnosis will therefore depend on the level of clinical suspicion and other contextual information (like age, smoking history, family history, travel history, etc.) and will usually become narrowed during the course of the diagnostic process. Other diseases should also be excluded that may give the impression of sarcoidosis but are non-granulomatous, such as lymphomas, other malignancies, and immune-mediated conditions like IgG4-related disease or auto-inflammatory syndromes like VEXAS syndrome, the latter being increasingly recognized as a novel entity [14].

When evidence of granulomatous inflammation is found, the differential diagnosis can be categorized into granulomatous disorders of either infectious or noninfectious origin. Table 2 provides a schema of these diagnoses in relation to the site of thoracic involvement. Infectious granulomas are often associated with necrosis, whereas typical sarcoid granulomas are not; i.e., they are non-caseating. However, it is important to note that presence or absence of necrosis in a biopsy is of relative importance. In a recent large-cohort study, it was shown that both presence or absence of necrosis in a biopsy specimen are possible in sarcoidosis [15].

Differential diagnosis of sarcoidosis requires customization, taking into account not only the individual’s clinical history and presentation but also risk factors, and can depend on geographic situation. Of note, due to migration and the increase of human travel activity over the past decades, infectious causes of granulomatous lung disease that used to be tied to certain continents can now also show up elsewhere (e.g., histoplasmosis in the Netherlands) [16,17].

Finally, as the diagnosis of sarcoidosis is never fully secure, it is advisable to always maintain a healthy degree of skepticism that an alternative diagnosis has been overlooked, especially when the clinical course of the disease gives rise to this [18].

## 8. Tools for Diagnosis

In patients suspected of pulmonary sarcoidosis, various tools are available to secure the diagnosis of sarcoidosis. None of the tests can be regarded as diagnostic proof alone. The extension and the nature of the tests will depend on the degree of ambiguity of the clinical presentation. Usually, a combination of tests lead to sufficient confidence that sarcoidosis may be diagnosed.

The first step is an assessment of epidemiological factors, notably the incidence of sarcoidosis and of alternative diagnoses in the region/country and exposure to risk factors (e.g., infectious, occupational, and environmental agents). Also, exposure to drugs taken for therapeutic or recreational purposes must be addressed. Family history is of importance, as approximately 10% of sarcoidosis patients report familial occurrence [19].

Subsequent investigations usually include (chest-)imaging, serum biomarkers, bronchoscopy with or without bronchoalveolar lavage, endo sonography, and/or pathologic evaluation of biopsy tissue. Each of these tests are discussed below with focus on their diagnostic value. Of note, to support clinicians as to the probability of sarcoidosis, especially in situations where biopsies might not be easy to perform, it might also be helpful to use clinical scores that support the likelihood of sarcoidosis in front of a compatible presentation [20,21].

### 8.1. Chest Imaging

The discovery of electromagnetic radiation and subsequently that of chest radiography made the early pioneers of sarcoidosis aware that the disease was much more than a skin, eye, and joint disease and that lungs and/or intrathoracic lymph nodes were the prime manifestation of the disease [22].

Based on chest radiography, thoracic sarcoidosis has classically been staged in four groups [23,24]. Stage I involves bilateral hilar lymph node enlargement (BHL); stage II shows BHL and pulmonary infiltration; in stage III only, pulmonary infiltration is found; and in stage IV, features of fibrosis, often with distortion of macroscopic lung architecture and calcifications, are demonstrated.

Although the staging has some prognostic significance (stage I: high likelihood of spontaneous resolution; stage II: spontaneous resolution possible; stage III: spontaneous resolution in rare cases; stage IV: permanent organ damage), it has many limitations. First, interobserver variability is poor, especially between stages with parenchymal involvement. Second, the stages suggest a relationship between disease severity and/or the order in which sarcoidosis may evolve. However, this is far from true, as a patient with stage I might seem to have mild disease but instead can suffer from severe cardiac involvement. Furthermore, although stage I on a chest radiograph is associated with high probability of resolution of intrathoracic lymphadenopathy after 1–2 years, the disease may nevertheless still evolve to progressive sarcoidosis in a minority of patients. Thus, instead of *stage*, the term radiographic *type* is more appropriate for use here [1].

**Figure 1:**
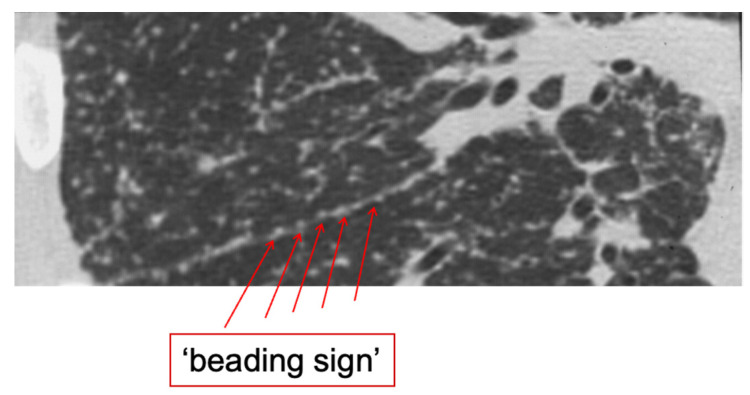
“Beading” along a fissure of the right lung in a patient with pulmonary sarcoidosis. Image source: Dr. H.W. van Es (Dept of Radiology, St. Antonius Hospital, Nieuwegein, The Netherlands).

Currently, HRCT is regarded as the most valuable tool for the diagnosis of pulmonary sarcoidosis. With this technique, characteristic features can be visualized, such as “beading” along fissures (Figure 1) and a peri-lymphatic micronodular pattern that, in combination with symmetrical nodal involvement and supportive clinical findings (see also Table 5 later on in this article), make sarcoidosis very likely [25]. Also, signs of fibrosis and different patterns of fibrosis can be identified more consistently on HRCT than on chest radiograph, including bronchial distortion, linear pattern, and cystic lung disease, which can be accompanied by honeycombing [26]. In addition, HRCT contributes in two other conditions. Firstly, HRCT is essential for establishing a confident diagnosis of progressive fibrosis in advanced pulmonary sarcoidosis, which may occur in approximately 15% of patients with advanced disease [27]. Also, HRCT can be useful for diagnosis of possible complications in pulmonary sarcoidosis, such as aspergilloma and pulmonary hypertension (by measuring pulmonary artery diameter) [28].

Imaging of pulmonary sarcoidosis is further discussed elsewhere in this issue.

### 8.2. Nuclear Imaging

Simultaneous uptake of (67)gallium (^67^Ga) in the salivary and lacrimal glands (panda sign) and intrathoracic lymph nodes (lambda sign) has shown to represent distinctive nuclear imaging patterns that are highly specific for sarcoidosis. In the 1990s, the combination of both panda and lambda sign or panda sign in combination with bilateral symmetrical hilar lymphadenopathy on chest X-ray has been suggested to obviate the need for invasive diagnostic procedures [29,30].

Today however, the use of ^67^Ga scanning in diagnostic evaluation of sarcoidosis has been outperformed by fluor-18-deoxyglucose (FDG) positron emission tomography (PET) scanning. It has been shown that FDG-PET is more sensitive than ^67^Ga imaging in the assessment of sarcoidosis activity. Furthermore, FDG-PET has demonstrated a very good inter observer agreement in contrast to ^67^Ga imaging [31]. Of note, the radiation dose is significantly higher for ^67^Ga imaging than for FDG-PET. In ^67^Ga imaging, the radiation dose is 18.5 mSv compared to 5.6–7.6 mSv for FDG-PET, depending on the patient’s weight [31].

It is not recommended to apply FDG-PET routinely in the diagnostic work-up of sarcoidosis, but in selected cases, it can be useful in identifying sites for biopsy or in differentiating extinguished fibrotic lesions from treatable inflammatory disease.

### 8.3. Serum Biomarkers

Recent evaluation of the diagnostic value of different serum biomarkers in sarcoidosis has revealed the best performance of serum angiotensin converting enzyme (sACE), soluble IL-2 (sIL-2R) receptor, and chitotriosidase (CTO). These markers stand out as the most useful diagnostic tools, with significant sensitivity and specificity, although none functions alone as a gold-standard biomarker [32]. The same markers also have significant value as monitoring tools after establishing a diagnosis, as change correlates with lung function improvement during methotrexate therapy [33]. A summary of test characteristics is given in Table 3. Of note, ACE diagnostic test performance can be significantly improved by performing genotype correction [34,35]. Further, none of the biomarkers mentioned is currently recommended for differential diagnosis by itself, although it seems plausible that a combination of different biomarkers might further improve sensitivity and specificity and become the standard of care in the future, but this needs further investigation [36].

### 8.4. Bronchoscopy

Bronchoscopy can reveal endobronchial lesions due to the mucosal involvement of sarcoidosis. These lesions are typically referred to as “cobble stone lesions” and reveal a high likelihood of finding granulomas upon biopsy.

Also, in the absence of visual lesions, there is chance of finding granulomas in random biopsies taken from the endobronchial mucosa of patients suspected of pulmonary sarcoidosis [37]. Even when the mucosa appears normal, biopsy of tissue at the first and secondary carinas is still positive in about 20–30% of patients [38].

Bronchoalveolar lavage (BAL) is a useful and safe procedure that is widely applied in the diagnostic evaluation of pulmonary sarcoidosis. Cytologic evaluation of BAL fluid shows lymphocytic alveolitis in 90% of patients and therefore contributes to the likelihood of diagnosis [39]. Also, a CD4/CD8 ratio >3.5 is generally regarded as supportive for the diagnosis [40]. However, no single feature in BAL is diagnostic proof of sarcoidosis. Only in an appropriate clinical setting does a CD4/CD8 ratio >3.5 provide a likely diagnosis of sarcoidosis with a specificity of 94% [2]. Additionally, relatively novel studies show that lower CD103 expression on CD4+ lymphocytes and markers identifying Th17.1 cells might have diagnostic value, but data are limited, and further studies are needed before clinical recommendations can be made [41].

Finally, the last important role of BAL to be mentioned is, of course, narrowing the differential diagnosis, e.g., by excluding (opportunistic) infections.

### 8.5. Endo Sonography

In most centers, endo sonography will by now have replaced mediastinoscopy as the standard procedure for intrathoracic nodal sampling in the diagnosis of pulmonary sarcoidosis. The latest guidelines justify the preference of endobronchial ultrasound (EBUS)-guided lymph node sampling (87% yield) over mediastinoscopy (98% yield) because it is safer for the patient and usually better tolerated [3]. Also, costs are generally lower for procedures such as EBUS that are performed in an endoscopy room compared with an operating room. International recommendations related to lymph node sampling in patients suspected of pulmonary sarcoidosis are given in Table 1.

Recently, EBUS transbronchial needle aspiration (TBNA) has been compared head-to-head with esophageal endoscopic ultrasound (EUS)-B fine-needle aspiration (FNA) for diagnosing sarcoidosis [42]. The results of this randomized clinical trial, including 358 patients from 14 hospitals in 9 countries, showed a similar granuloma detection rate of mediastinal/hilar nodes in patients suspected of pulmonary sarcoidosis (Scadding stage I/II). The granuloma detection rate was 70% for EBUS-TBNA and 68% for EUS-B-FNA. The authors concluded that both diagnostic tests can be safely and universally used in patients suspected of sarcoidosis [42]. However, EBUS has an additional advantage over EUS, as it allows adding transbronchial biopsy when lymphadenopathy is accompanied by the radiographic findings of parenchymal disease or endobronchial biopsy when mucosal abnormalities are noted and/or BAL during endoscopy, which further increase the diagnostic yield [3].

### 8.6. Peripheral Lung Biopsy

There are different techniques to collect tissue from the peripheral lung parenchyma for the diagnosis of sarcoidosis.

A summary of different lung tissue sampling procedures, including diagnostic yield, is given in Table 4. The choice of method will often also depend on the possibilities and experience within a particular center.

Of note, only in very few cases suspected of sarcoidosis will performing surgical lung biopsy for confirmation of the diagnosis be necessary. In such cases, it may be advisable to first weigh the advantages and disadvantages of a surgical lung biopsy in a multidisciplinary discussion, with special attention to the diagnostic added value and therapeutic consequences.

As mentioned above, non-caseating granulomas are the pathological hallmark of sarcoidosis. Typical for sarcoidosis is that the granulomas are well formed, without significant surrounding lymphoid infiltrate. The granulomas are discrete and compact (also called “naked granulomas”). Although this type of granuloma is characteristic for sarcoidosis and sometimes referred to as “sarcoid granuloma”, it may also be found in other conditions such as Blau’s syndrome, foreign material, drugs, secondary syphilis, common variable immune deficiency, and chronic granulomatous disease [37,43]. Also, sarcoid granulomas typically contain multinucleated giant cells, sometimes containing cytoplasmic inclusions such as asteroid and Hamazaki–Wesenberg and Schaumann bodies [2,44].

The other key feature of sarcoid granulomas is their anatomic distribution. Sarcoid granulomas in the lung are characteristically found along lymphatics, around the bronchovascular bundles, in the interlobular septa, and on the pleural surface. The number of granulomas in each of these locations may vary, but generally, they are more abundant around the bronchovascular bundles. Sarcoid granulomas and/or giant cells may also be found around and sometimes in the wall of pulmonary arteries or veins with a weak-to-absent inflammatory infiltrate and without necrosis of the vessel. The latter pathological finding is, however, not usually clinically associated with pulmonary hypertension or veno-occlusive disease [37].

As finding necrosis in relation to granulomas should always raise high suspicion of an infectious granulomatous disease, the presence of some necrosis in the granulomas of patients with confirmed sarcoidosis has been described in up to 20% of biopsies [15]. Necrotic foci generally consist of small foci of fibrinoid (“rheumatoid-like”) necrosis punctuating occasional granulomas, whereas larger areas of fibrinoid, infarct, or suppurative (“GPA-like”) necrosis may be rarely seen [45]. When necrosis is particularly prominent, entity-necrotizing sarcoid granulomatosis may be considered. In general, however, the presence of necrosis in granulomas should always raise the possibility of infection, and a diagnosis of necrotizing sarcoid granulomatosis, which is probably an unusual variant of sarcoidosis, should not even be considered until an infection has been unconditionally excluded.

### 8.7. Pulmonary Function Testing

Pulmonary function testing is also central to the evaluation of patients suspected of sarcoidosis but in whom results are not contributing to diagnosis, although they reveal important information on the severity and/or progression of disease and can determine decisions on invasive diagnostic procedures, as mentioned above. Typically, in pulmonary sarcoidosis, all kinds of abnormal ventilatory patterns are possible, including mixed ventilatory defects, which have recently been reported to occur in approximately 10% of patients [46]. Also, it is important to note that pulmonary functions tests may not reflect disease activity or symptom burden. Pulmonary function in sarcoidosis is further discussed elsewhere in this issue.

## 9. Diagnostic Approach

**Figure 2:**
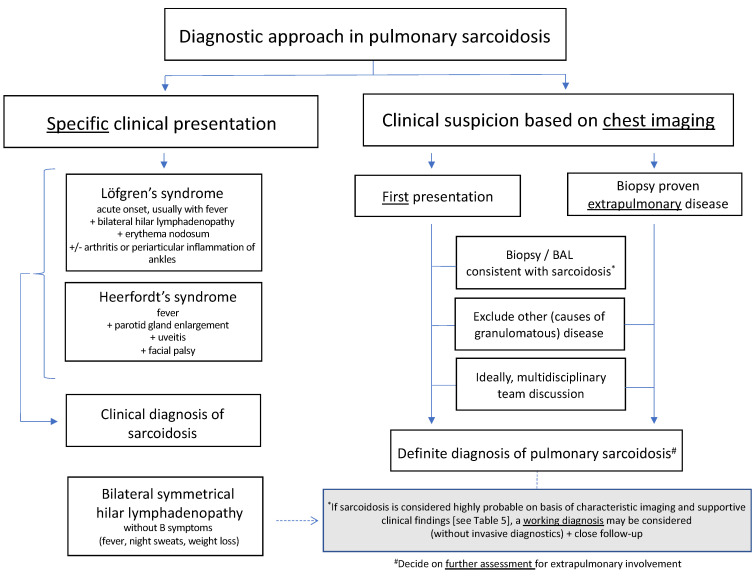
Algorithm for the diagnostic approach in pulmonary sarcoidosis.

An algorithm for the diagnostic approach in pulmonary sarcoidosis is given in Figure 2. It consists of a multistep process that usually starts with the clinical suspicion based on chest imaging. In some cases, the disease can be diagnosed clinically, without performing a tissue biopsy (left side of the figure), especially when there is no need for systemic treatment. Otherwise, cytologic or histologic evidence of granulomatous inflammation and exclusion of alternative causes are required for a confident diagnosis, ideally after multidisciplinary team discussion. In both cases, compliance to the diagnostic criterium on the exclusion of alternative causes of granulomatous disease such as tuberculosis or fungal infection and using the appropriate methods are of utmost importance.

In case sarcoidosis is regarded highly probable on the basis of collected clinical data, including supportive findings such as elevated serum ACE and/or sIL2R and others (Table 5), it is not uncommon, especially in centers with expertise in sarcoidosis, to decide on a “working diagnosis of sarcoidosis” and to refrain from further invasive diagnostic procedures for the patient, especially when patients are not threatened by organ failure or organ damage due to sarcoidosis and when indication to start immunosuppressive therapy is absent at that time, or the patient is frail. A working diagnosis should preferably be agreed upon after discussion in MDT [7].

The ultimate goal of the diagnostic process is to rule out all diagnoses other than sarcoidosis that are consistent with the clinical situation. In some patients, a definite diagnosis may require the continuous gathering of information during follow-up. After diagnosis, a healthy degree of skepticism remains indispensable, especially in the case of an unexpected course of disease during follow-up. In that situation, additional investigations might be needed with reconsideration of the diagnosis.

## 10. Multidisciplinary Team

As sarcoidosis is defined as multisystem disease, it would be plausible to involve clinicians of other disciplines than pulmonology in the diagnostic process. The value of multidisciplinary team (MDT) discussion has already been scientifically illustrated and evaluated in ILD [47] and subsequently implemented particularly in the diagnostic guidelines of idiopathic pulmonary fibrosis [48,49,50]. In addition, MDT discussion in diagnosis of connective tissue disease–ILD (CTD-ILD) has been recommended [51]. However, until now, the literature is still lacking in recommendations for the particular case of sarcoidosis.

Nevertheless, many clinicians working in the field of sarcoidosis find an MDT discussion to be of added value, providing a momentum for intra- and interdisciplinary-supported diagnosis or generating new diagnostic considerations. Additionally, MDT discussion may next contribute to peer support for complex treatment decisions, which is especially important in the absence of guidelines with high-quality evidence recommendations. The implementation of MDT discussion in care pathways for sarcoidosis is therefore an important criterium for the evaluation of patient-centered care for sarcoidosis in expert centers across Europe (https://health.ec.europa.eu/european-reference-networks/overview/evaluation-european-reference-networks_en, accessed on 17 September 2023).

**Figure 3:**
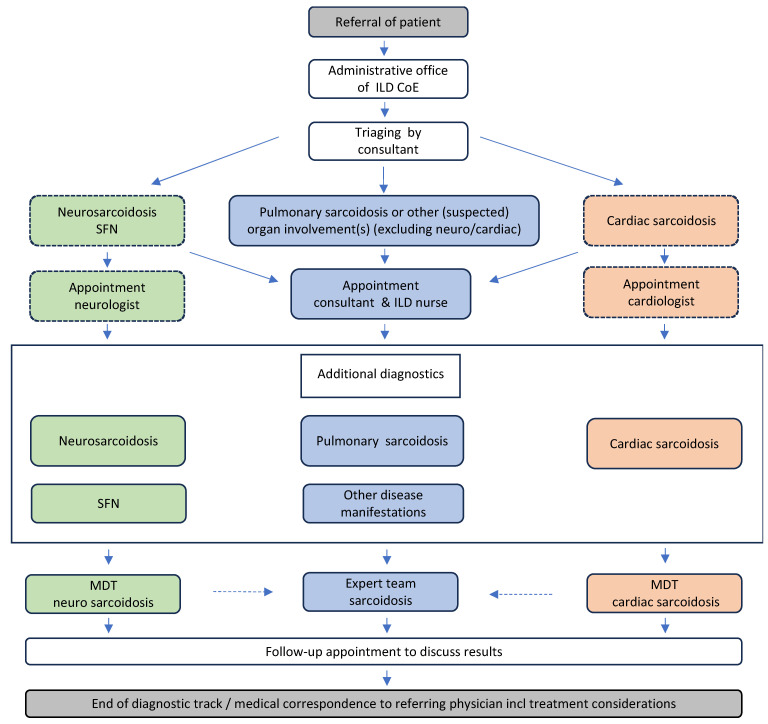
Module of the care pathway for sarcoidosis showing the diagnostic track of (suspected) patient referred to St. Antonius ILD and Sarcoidosis Center of Excellence Nieuwegein, The Netherlands. Definition of abbreviations: CoE, center of excellence; ILD, interstitial lung diseases; MDT, multidisciplinary team; SFN, small fiber neuropathy.

In the author’s ILD center of excellence, all patients that are referred with (suspicion of) sarcoidosis receive a standard work-up for diagnosis according to the local care pathway for sarcoidosis, including MDT discussion, depending on the type of major organ involvement (Figure 3).

Nonetheless, in general, not all patients have the opportunity to consult a center specialized in ILD. It is, however, the author’s experience that offering MDT conferences (either virtual or as a review service) for external patients can play a valuable role in the diagnostic decision and care of these patients. In this way, centers of excellence facilitate greater and more rapid access to sarcoidosis expertise.

## 11. Perspective

With ongoing advances in biomolecular technology and the development of artificial intelligence, it is likely that novel diagnostic tools will appear. With no doubt, these will change the methods of diagnosing sarcoidosis in the near future.

Interesting new developments in the field of chest imaging have recently been published. Photon-counting CT (ultra-HRCT) has been shown to improve image quality for visualization of certain ILD features, such as traction bronchiolectasis and micro-nodules. This technical advance not only results in lower radiation exposure but may also enhance the diagnosis and prognosis of pulmonary sarcoidosis and ILD in the near future [52].

An intriguing example of the potential of omics in establishing a diagnosis of sarcoidosis was recently found in the eNose study (SpiroNose) [53]. Based on analysis of exhaled breath patterns, the eNose technology significantly differentiated sarcoidosis patients from healthy controls as well as from patients with hypersensitivity pneumonitis. Further research is warranted to understand and prove the value of this non-invasive novel technology.

Finally, also research of new and especially combinations of biomarkers is regarded as a promising direction in the field of diagnosis and management of sarcoidosis and might lead to an improved standard of care in the future [36].

## 12. Concluding Remarks

The diagnosis of pulmonary sarcoidosis requires sufficient knowledge and experience with the disease, a sharp clinical eye, and a healthy dose of suspicion. Unfortunately, until now, no single, simple diagnostic test has been available. A systematic and multidisciplinary approach, preferably implemented in a local care pathway for sarcoidosis and including MDT discussion, currently provides the best guarantee for establishing the right diagnosis. In this context, based on ATS and BTS recommendations, increased specificity of CT features, and/or cases of acute presentation with specific symptoms, it might also be justifiable to refrain from further invasive procedures and follow-up for the patient.

## Figures and Tables

**Table 1:** Summary of current evidence-based recommendations on lymph node biopsy in patients suspected of sarcoidosis and presenting with mediastinal and/or hilar lymphadenopathy.

Clinical Context	Recommendation	Level of Evidence	Remark Experts
In patients for whom there is a high clinical suspicion for sarcoidosis (e.g., Löfgren’s syndrome, lupus pernio, or Heerfordt’s syndrome)	Lymph nodes sampling is not suggested (conditional recommendation)	Very low-quality evidence	Patients who do not undergo lymph node sampling require clinical follow-up
For patients presenting with asymptomatic, bilateral hilar lymphadenopathy			No recommendations for or against obtaining a lymph node sample can be made
For patients with suspected sarcoidosis and mediastinal and/or hilar lymphadenopathy for whom it has been determined that tissue sampling is necessary *	Endobronchial ultrasound (EBUS)-guided lymph node sampling, rather than mediastinoscopy, as the initial mediastinal and/or hilar lymph node sampling procedure is suggested (conditional recommendation)	Very low-quality evidence	

Adapted from Crouser et al. [3]. * Criteria are (1) the desired diagnostic certainty, especially when an alternative diagnosis is reasonably possible; (2) the consideration of possible immunosuppressive treatment; and (3) when there is lack of skin and/or peripheral lymph node findings for a less risky and less invasive method of tissue sampling.

**Table 2:** Differential diagnoses of pulmonary sarcoidosis, related to site of thoracic involvement.

Site of Thoracic Involvement	Infectious Granulomatous Diseases	Non-Infectious Granulomatous Diseases
Lung parenchyma	Tuberculosis	Hypersensitivity pneumonitis (many causal antigens)
	NTM infections	Chemical induced granulomatosis (e.g., beryllium, aluminum, zirconium, silica, and talc)
	Histoplasmosis (very rare in Europe)	Drug-induced granulomatosis (e.g., TNF-alpha antagonists, immune checkpoint inhibitors, targeted therapies, and interferons)
	Parasitic infections (very rare, e.g., leishmaniosis, paragonimiasis, and schistosomiasis), occurring mainly in endemic countries	Aspiration pneumonia with foreign body granulomatosis
	Viral infections (very rare, e.g., varicella zoster and cytomegalovirus), mainly in immunocompromised patients)	Vasculitis, CTD, and inflammatory disease (e.g., GPA, EGPA, NSG, ILD in Sjogren’s syndrome, and Crohn’s disease)
	Other infections (very rare, e.g., Whipple’s disease, cryptococcosis, coccidioidomycosis, and mucormycosis), mainly in immunocompromised patients	Immune deficiency granulomatosis (e.g., granulomatous-associated CVID and CGD)
		Genetic disorders: Blau syndrome
		Malignancy-associated granulomatosis (e.g., cancer and lymphoma)
		Other proliferative disorders (e.g., LCH, ECD, and lymphomatoid granulomatosis)
Thoracic lymph nodes	Tuberculosis	Sarcoid-like reaction (especially occurring in linkage to malignancies but also in rare occasions of hypersensitivity pneumonitis and CTD such as Sjogren’s syndrome)
	NTM infections	Chemical-induced granulomatosis (e.g., beryllium, aluminum, zirconium, silica, and talc)
	Histoplasmosis (very rare in Europe)	Drug-induced granulomatosis (e.g., TNF-alpha antagonists, immune checkpoint inhibitors, targeted therapies, and interferons)
	Other infections (very rare, e.g., Whipple’s disease and fungal infections)	Immune deficiency granulomatosis (e.g., granulomatous-associated CVID and CGD)
		Malignancy-associated granulomatosis (e.g., cancer and lymphoma)

Definition of abbreviations: CGD, chronic granulomatous disease; CTD, connective tissue disease; CVID, common variable immune deficiency; ECD, Erdheim–Chester disease; EGPA, eosinophilic granulomatosis with polyangiitis; GPA, granulomatosis with polyangiitis; ILD, interstitial lung disease; LCH, Langerhans cell histiocytosis; NSG, necrotizing sarcoid granulomatosis; NTM, nontuberculous mycobacteria; TNF, tumor necrosis factor. The differential diagnosis should be prioritized on the basis of the individual’s clinical history and presentation and can depend on geographic location.

**Table 3:** Serum biomarkers for diagnosing sarcoidosis.

Biomarker as a Diagnostic Tool	Sensitivity, %	Specificity, %	First Author [Ref.]
sACE	20–90.5	47–89.9	Nguyen, Eurelings, Uysal, Csongrádi, Lopes, and Ungprasert
sIL-2R	47–94.4	90.4	Nguyen, Eurelings, Keijsers, Schimmelpennink, and Miyata
CTO	82.5–88.6	70–92.8	Popevic, Enyedi, and Bargagli

Definition of abbreviations: sACE, serum ACE; sIL-2R, soluble IL-2 receptor; CTO, chitotriosidase. Adapted Table 2 from Korenromp I.H.E., Maier L.A., Jan C. Grutters J.C. Sarcoidosis: Serum and Imaging Biomarkers. In *Sarcoidosis (ERS Monograph)*; Bonella, F., Culver, D.A., Israël-Biet, D., Eds.; European Respiratory Society: Sheffield, UK, 2022; pp. 107–121 (https://doi.org/10.1183/2312508X.10031720), reproduced with permission of the © ERS 2023 [32].

**Table 4:** Different tissue sampling procedures and their diagnostic yield for the diagnosis of pulmonary sarcoidosis.

Method	Diagnostic Yield	Invasiveness	Granuloma	Lymphatic Pattern	Comments
Conventional transbronchial biopsy	High (up to 70–80%)	Intermediate/high	Yes	Yes	Sarcoid granulomas and lymphatic pattern may be appreciated; serial sections may be very helpful in highlighting granulomas when absent in the first slides
Transbronchial cryobiopsy	Very high (up to 100%)	High (10–15% pneumothorax; occasionally hemorrhagic events)	Yes	Yes	Very helpful in case of negative results from more conventional procedures and to avoid open-lung biopsy
Surgical lung biopsy	Very high (100%)	Very high (patients should be carefully selected); non-intubated, “awake” biopsy reduces complications	Yes	Yes	Limited to very challenging cases when transbronchial procedures failed to demonstrate granulomas (i.e., chronic form with hyaline sclerosis replacing granulomas and mimicking other ILDs)

Adapted Table 1 from Rossi G, Farver C. Sarcoidosis: pathological features and differential diagnosis. In *Sarcoidosis (ERS Monograph)*; Bonella, F., Culver, D.A., Israël-Biet, D., Eds.; European Respiratory Society:Sheffield, UK, 2022; pp. 107–121 (https://doi.org/10.1183/2312508X.10031720), reproduced with permission of the © ERS 2023 [37].

**Table 5:** Supportive and not-supportive clinical findings for likelihood of pulmonary sarcoidosis.

Clinical Data	Supportive	Not Supportive
Demographics	African American	Age < 18 years; >80 years
	Northern European	
Medical history	Family history of sarcoidosis	
	Non-smoker	
	History of unexplained fatigue and/or pain	
	Symptoms involving two or more organs	
	Specific combinations, e.g., lung and eyes; lung and skin	
	History of kidney stones	
Extrapulmonary disease potentially related to sarcoidosis	Uveitis, erythema nodosum (small fiber), neuropathy, etc.	
Disease course		Rapid progressive (diffuse) lung disease (days to few week) +/− respiratory failure
Laboratory results	Increased serum sACE	
	Increased sIL-2R	
	Increased CTO	
	Lymphopenia	
	Increased serum calcium	
	Hypercalciuria	
	Decreased 25-hydroxyvitamin D/increased 1,25-hydroxyvitamin D	
BAL findings	Lymphocytosis	
	Increased CD4+/CD8+ ratio	
	Decreased CD103+CD4+/CD4+ ratio	

Definition of abbreviations: sACE, serum ACE; sIL-2R, soluble IL-2 receptor; CTO, chitotriosidase; BAL, bronchoalveolar lavage.

## Data Availability

Not applicable.
